# The Positive Effect of Psychotherapy in the Treatment of an Erosive Oral and Dermal Lichen Planus Case

**DOI:** 10.1002/ccr3.9716

**Published:** 2025-01-09

**Authors:** Zana Fuad Noori, Mohammed Khalid Mahmood, Pinar Yaseen Raof, Handren Ameer Kurda, Balen Hamid Qadir, Didar Anwar Abdulrahman, Mohammed Aso Abdulghafor, Mohammed Taib Fatih, Basoz Khalid Hama Noori

**Affiliations:** ^1^ Oral Biology Department, Dentistry College American University of Iraq‐Sulaimani AUIS Sulaymaniyah Iraq; ^2^ Odotology Department Aix‐Marseille University, CNRS, EFS, ADES Marseille France; ^3^ College of Dentistry Sulaimani University Sulaymaniyah Iraq; ^4^ Department of Dentistry Komar University of Science and Technology Sulaymaniyah Iraq; ^5^ Clinical Psychology Department Koya University Koya Iraq

**Keywords:** erosive lichen planus, lichen planus, oral lichen planus, psychotherapy

## Abstract

Lichen planus (LP) is an autoimmune disease that may affect the oral cavity and the skin, and it has the potential to change to malignancy. In this paper we report a LP case in a 42‐year‐old male patient in which anxiety and depression were apparently the only possible risk factors of LP. Due to this apparent comorbidity and the risk of not responding well to conventional medication (topical and systemic corticosteroids) or the risk of relapse in the case of ongoing stress, we decided to integrate psychotherapy (without psychotropic drugs) as an adjunct into the management strategy. Psychotherapy sessions, reassuring the patient and stress alleviation, proved to be a very useful alongside the standard corticosteroid medication.

**Summary**

- This paper shows that stress and its associated anxiety and depression have the potential to be the only possible cause for the development of oral lichen planus.
- Psychotherapy targeting stress alleviation proved to be fruitful in conjunction with conventional treatment of our patient.

## 1 Introduction

Lichen planus (LP) is a chronic inflammatory disease of the oral mucosa and the skin, which is of autoimmune origin. LP can be clinically classified into nonerosive (reticular, plaque, atrophy, and blister) and erosive type. LP has the potential to involve the skin and mucous membranes of the oral, vulvovaginal, esophageal, laryngeal, and conjunctival regions [1].

Symptoms of oral LP (OLP) include pain, ulceration, dysphagia, and difficulty in speaking and chewing. The erosive type of OLP has been defined by the WHO as potentially malignant [2].

The prevalence of erosive OLP in clinical practice is 0.1%–4% [3] and 1.01% worldwide [4]. The buccal mucosa is usually affected in 80%–90% of OLP cases. OLP is known to usually affect the middle‐aged men and women, and some researchers have documented a higher incidence rate in women with a ratio of 2:1. It is a serious disease and about 1% of the cases may change to malignancy [3].

Although the causes of OLP are not yet clearly understood and sometimes are of unknown origins; there have been reports of several risk factors, diseases, and conditions such as autoimmune diseases, stress/anxiety, viral infections especially hepatitis C virus, dyslipidemia, and internal malignancies [5].

Stress can be defined as a state of threatened homeostasis. The term stress has become popular and well known in the last two decades. Today, almost every adult individual seems to have had a negative encounter with it at some point in their lives [6]. Stress has been linked to participate in the initiation and exacerbation of several diseases including autoimmune diseases. Autoimmune diseases are thought to be multifactorial. Besides genetic, environmental, hormonal, and immunological factors, psychological stress has also been found to be positively linked to autoimmune diseases [7].

In this paper we report an LP case in a 42‐year‐old male patient in which anxiety and depression were apparently the only possible risk factors of LP. Due to this apparent comorbidity and the risk of not responding well to conventional medication (topical and systemic corticosteroids) or the risk of relapse in the case of ongoing stress, we decided to integrate psychotherapy (without psychotropic drugs) as an adjunct into the management strategy. Psychotherapy sessions, reassuring the patient and stress alleviation, proved to be a very useful alongside the standard corticosteroid medication.

## 2 Case Presentation

**FIGURE 1:**
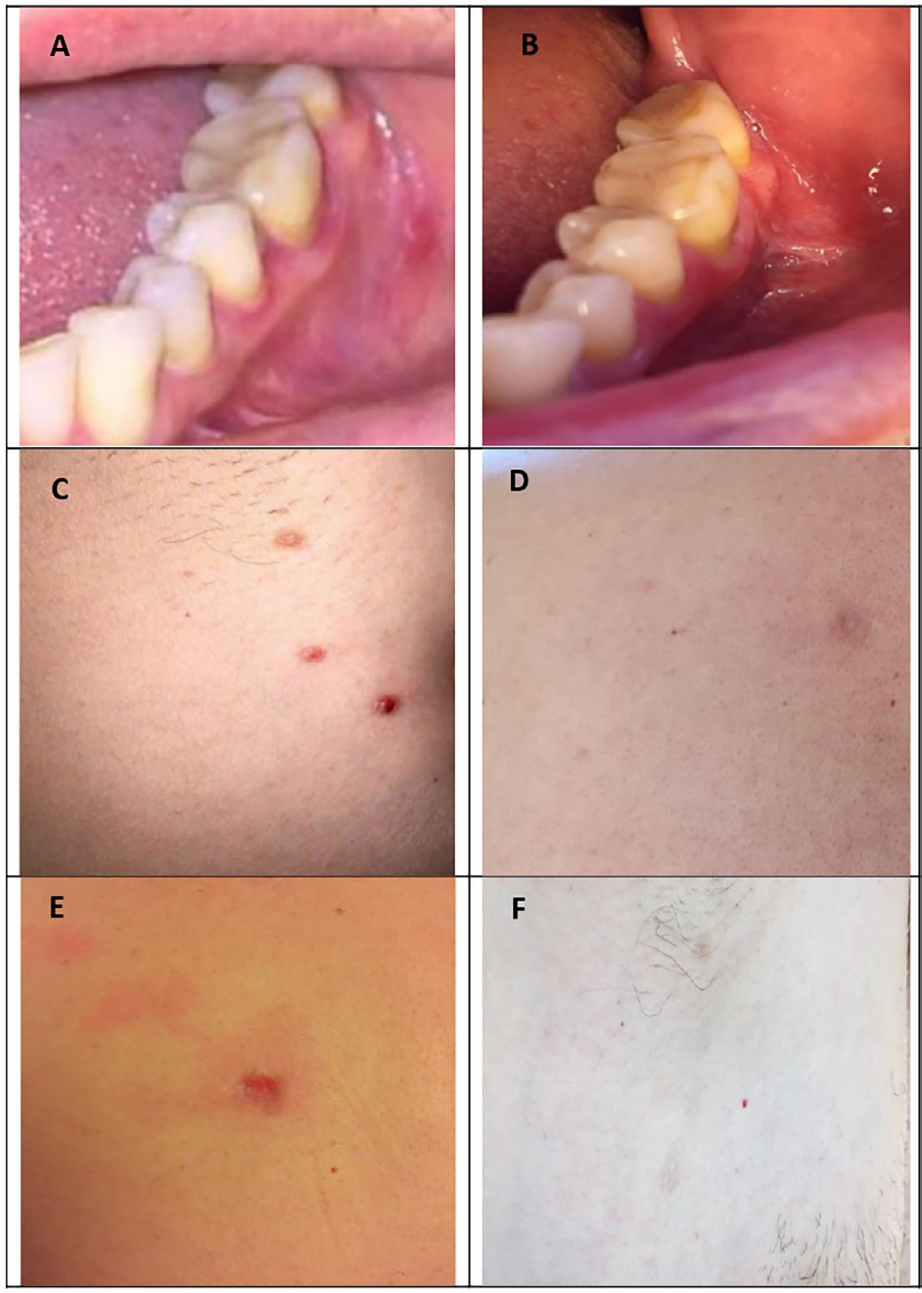
(A, B) The lesion on lower left buccal mucosa before and after treatment. (C, D) Lesions on his back before and after treatment. (E, F) Lesions on his chest before and after treatment.

**FIGURE 2:**
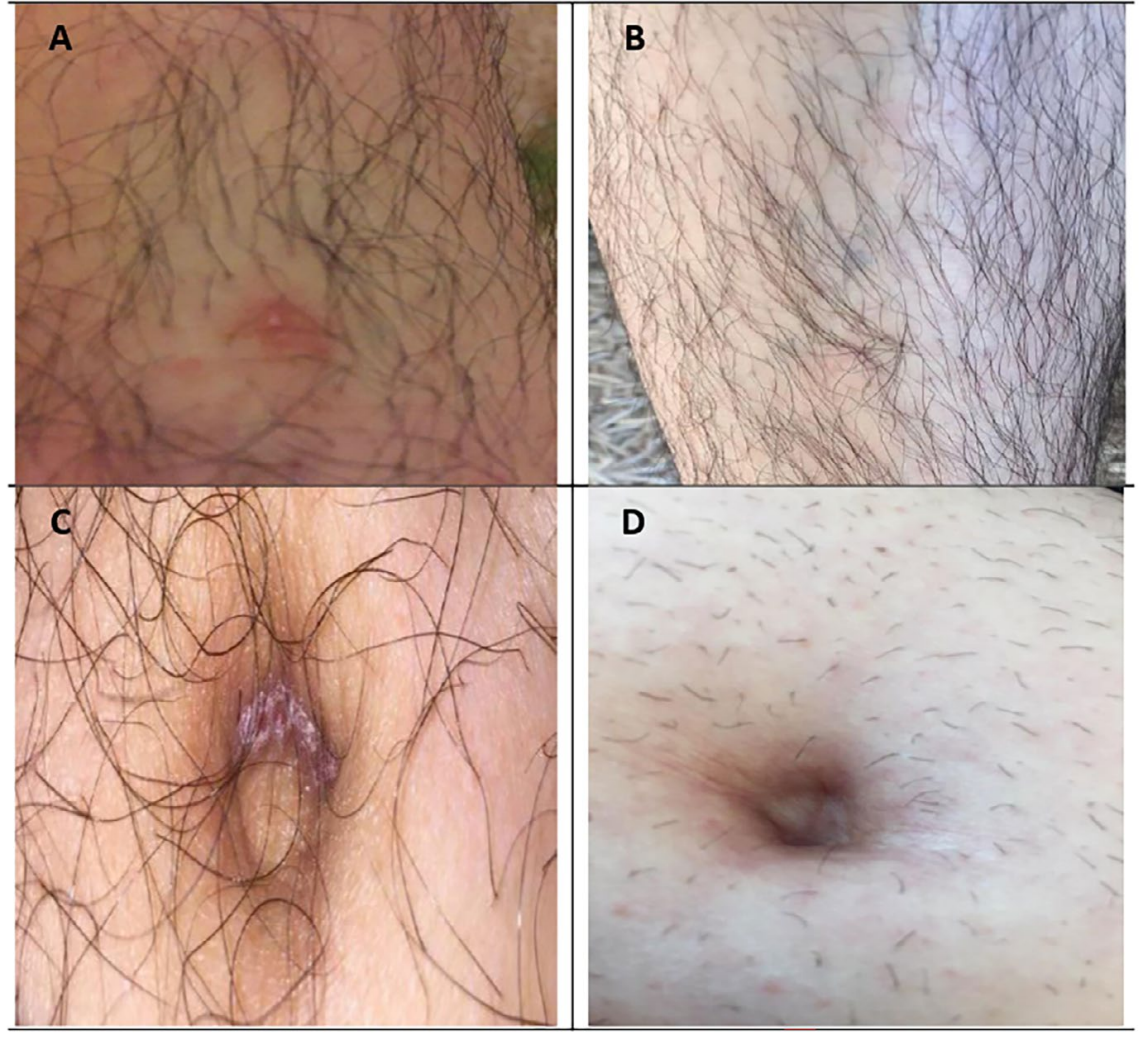
(A, B) Lesions on his legs before and after treatment. (C, D) The lesions on his abdomen/navel before and after treatment.

A 42‐year‐old male patient visited Piramerd Oral & Dental Specialty Center and complained of persistent white and red lesions with ulcerations for 6 months duration in the mandibular left buccal mucosa below the 1st and 2nd molars. The lesions were associated with pain at the lower left buccal mucosa and the vestibular fold. The lesions were not confined only to oral cavity; ulcers also erupted on his back, chest, legs, and abdomen (Figures 1 and 2). All of the lesions were associated with persistent pain. The patient stated that his oral lesions appeared first followed by his skin lesions. The patient complained from dysphagia and burning sensation in his oral cavity, stomach, and esophagus.

The patient's history was taken. He reported no history of any chronic disease or any form of medication therapy. The family history also revealed no association with the disease. The patient was non‐smoker and he was not consuming alcohol. He was otherwise healthy and physically fit. However, the patient reported that he has been suffering from a strong tide of depression and stress; he also reported that he has been suffering from insomnia and sexual incontinency.

## 3 Investigation

Based on the careful clinical examination, our differential diagnosis included systemic pemphigus, squamous cell carcinoma (SCC), and LP. On the same day of his first visit, biopsy was taken from the left lower buccal mucosa in the vestibular fold just below the 1st and 2nd molars.

## 4 Diagnosis and Treatment

The biopsy was sent for histopathological examination and the diagnosis was erosive LP. After the final diagnosis, the treatment was initiated immediately. The patient was put on the following treatment plan: Prednisolone table 5 mg once daily, Prednisolone syrup as mouthwash three times daily, and Betamethasone ointment for his body lesions.

The patient was sent to a psychologist for the management of his stress. The Perceived Stress Scale (PSS) is used as an instrument to measure the stress level. This tool makes it easier to comprehend how various circumstances impact a person's emotions and perceived stress. This scale asks about thoughts and feelings throughout the previous month. PSS score can range from 0 to 40. A higher score means a higher perceived stress level. Before the treatment, the patient's PSS scale was 36.

A weekly consultation meeting was scheduled, during which psychoanalytic techniques were primarily used to address internal conflicts and cognitive behavior therapy was used to modify his feelings toward certain individuals and situations. The patient was instructed to do exercise, reduce his stress as much as possible, get good sleep, and have a healthy diet to boost his immunity. The patient was then put on a follow‐up plan.

## 5 Outcome and Follow‐Up

### 5.1 Follow Up After 1 Week

The patient reported decreased redness and pain in his oral lesions. The dermal lesions showed no sign of healing and were still painful. He was still stressed and worried about his health condition. The dose of the Prednisolone tab. was increased to 5 mg twice daily. The Prednisolone syrup as mouthwash and Betamethasone ointment were again prescribed. PSS scale was decreased to 29.

### 5.2 Follow Up After 2 Weeks

The oral lesions were the same as the first week (decreased redness). However, pain from these oral lesions had subsided totally. His dermal lesions were less painful. He reported that the burning sensation in his stomach and esophagus had subsided too. Two intra‐lesional injections of Triamcinolone acetonide 40 mg (once each week) were prescribed and the first injection was administered. This was done to increase the speed of the healing process. The patient was kept on Prednisolone table 5 mg twice daily and his steroidal mouthwash and ointment. PSS scale was decreased to 18.

### 5.3 Follow Up After 3 Weeks

The patient's dermal lesions all healed, except for his back lesion. The 2nd intra‐lesional oral injection of Triamcinolone acetonide 40 mg was administered. The pain remained only in his back lesion. The patient was kept on steroidal mouthwash and ointment. The patient was still advised to continue taking his 5 mg Prednisolone tablet twice daily. The patient was very positive and seemed less stressed. PSS scale was decreased to 15.

### 5.4 Follow Up After 4 Weeks

Intra‐oral lesions totally healed by the fourth week. Skin lesions disappeared including his back lesion (Figures 1 and 2). The patient reported that he has returned to his normal sexual life. Moreover, he also reported an increase in the quality of his sleep. The prednisolone tab was then tapered down to 5 mg/day for 1 week, then 5 mg every other day for another week, and then it was completely stopped. The patient was instructed to do exercise, take vitamin C supplements, and have a healthy diet. PSS scale was decreased to 10.

### 5.5 Follow Up After 6 Months

**FIGURE 3:**
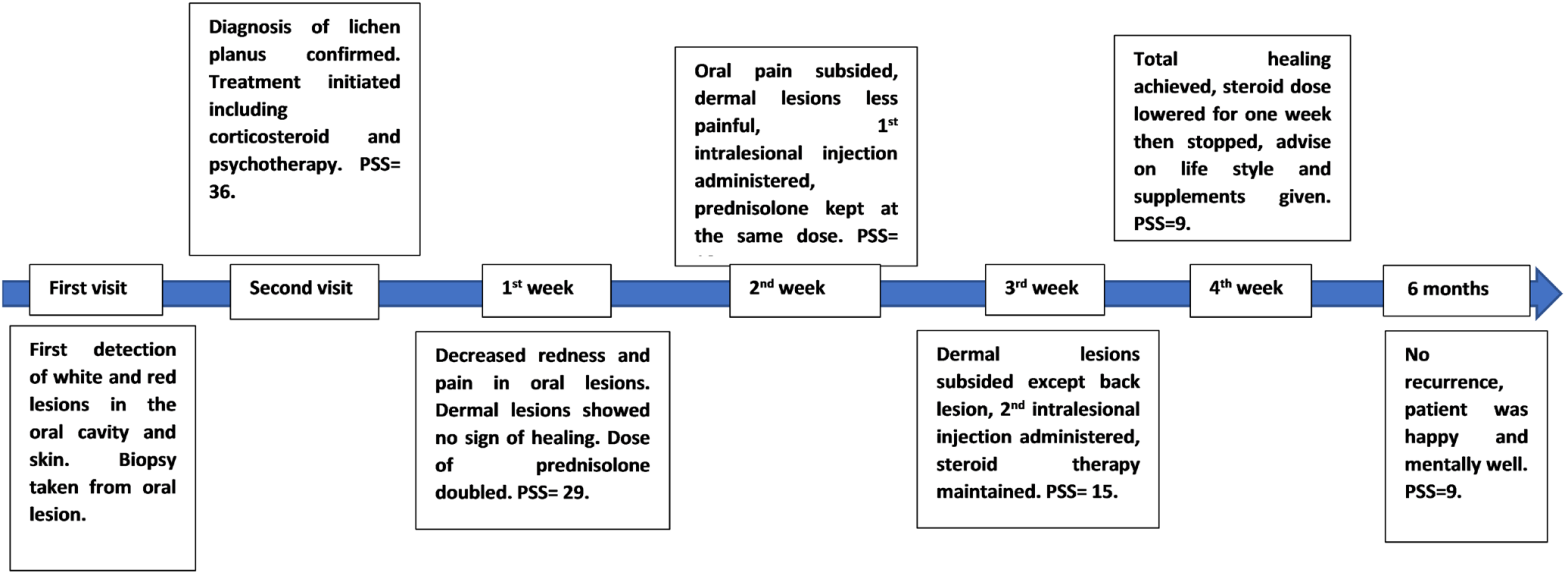
Disease timeline of the patient from the beginning until recovery.

The patient reported total recovery and no new lesion eruption neither in his oral cavity nor anywhere else on his skin during that time. He was in good health and was mentally and physically well. The PSS scale was decreased to 9. Figure 3 shows the disease timeline of the patient from the beginning until recovery.

## 6 Discussion

LP is a chronic inflammatory disease of the oral mucosa and the skin, which is of autoimmune origin. The erosive type is relatively more important as compared to the other types as it can be severely painful and affect the quality of life of the patient [8]. It has been reported that patients with erosive OLP have a poor quality of life due to the associated dysphagia, difficulty in speaking and chewing, ulceration, and pain. OLP is also known to cause psychological and social issues for the patient and even in some cases the walking of the patient is also impaired [9]. In our case, the condition was negatively impacting the quality of life and interfering with the usual daily activities through dysphagia, burning sensation in the oral cavity, stomach, and esophagus, insomnia, and sexual incontinency.

Concomitant dermal and mucosal nature of LP is important to consider. In a study with 99 LP patients, 39 of them (39.4%) had dermal lesions along with their oral lesions [10]. In another study, 87 patients with OLP were examined and 36 of the patients (40%) had dermal lesions in addition [11]. About 7%–10% of LP patient have nail involvement [10, 11]. Our case had concomitant oral and dermal lesions without any signs of nail involvement.

Stress, when left unattended and not managed, will lead to a state of anxiety. When it continues for a long period of time it may also cause depression. Depression prevents the individual from coping with stress. Thus, a cycle is established, where depression causes more stress and the more the stress, the more depressive the individual gets [6].

Anxiety disorder prevalence worldwide varied from 0.9% to 28.3% based on 87 research conducted in 44 countries, while past‐year prevalence varied from 2.4% to 29.8% [12].

In general, stress has been linked with several diseases. Stress is found to be directly linked with several pathophysiological processes linked to neurodegenerative diseases including Alzheimer's disease [13] and multiple sclerosis [14]. Stress is also found as a contributing factor to several other well‐known diseases and conditions such as hypertension, atherosclerosis, diabetes, addiction, depression, anxiety, and even infertility [15].

Moreover, stress is also found to be a contributing factor to autoimmune diseases. A substantial correlation was found between exposure to a stress‐related problem and an elevated chance of developing later autoimmune diseases, including Sjogren syndrome, psoriasis, Addison's disease, vitiligo, Crohn's disease, ulcerative colitis, and rheumatoid arthritis [7]. Some studies have claimed that up to 80% of the cases of autoimmune diseases have reported emotional stress before the onset of the disease [16].

Stress and anxiety, together with other precipitating factors, participate in causing the OLP, then the developed OLP will even cause more stress and anxiety for the patient, thus creating a vicious cycle. Comparing OLP to healthy controls, a systematic review and meta‐analysis conducted by Li, He, Hua [17] revealed a strong correlation between OLP on the one hand and stress, anxiety, and depression on the other hand. In addition, specific personality traits and sleep disturbances also impacted OLP patients. According to a review, stress can cause anxiety and depression in postmenopausal women, which are common causes of LP [18]. According to a different study, 45% of the 274 oral LP patients exhibited anxiety, sadness, or stress, and 48% of them had mental health issues [19]. When considered collectively, these and similar findings suggest that OLP and its relapses may be caused by stress, anxiety, and depression. Our case was in agreement with the literature since stress was the only apparent and plausible factor in an otherwise healthy individual.

The standard treatment option for OLP includes the use of topical and systemic steroids. Prednisolone, in particular, is the most widely used medication due to its effectiveness, low rates of hypersensitivity, and great patient compliance [1]. We used the same regimen (a mixture between topical and systemic corticosteroids) for our case.

Besides these conventional medications, psychotropic drugs have also been used in the literature as adjunct therapy modalities. In some cases, the patients do not respond to the conventional LP medications, therefore psychotropic drugs are needed to induce complete recovery. For example, psychotropic medications were found to be helpful in the long run in managing oral symptoms in LP patients who were not responding to traditional immunosuppressive therapy in a research involving 28 cases of reticular OLP [20]. The combination of psychotropic medication therapy and standard treatment techniques was successful in decreasing the size of the lesions in an investigation including 46 individuals with OLP and psychiatric diseases, but it had no discernible impact on the symptoms of the participants [21].

In our case, due to the obvious presence of stress as comorbidity, integrating a psychological aspect in the management and treatment of the case is decided. However, in the beginning only psychotherapy sessions were started. The intention was to preserve the usage of psychotropic drugs for the next stage if needed. Thankfully, only the psychotherapy was sufficient to reach a complete recovery. Psychotherapy, especially cognitive behavior therapy (CBT), has shown to be particularly successful in treating insomnia and chronic pain [22]. People with depressed symptoms, exhaustion, and sleep deprivation showed significant improvements with CBT intervention, according to research by Wilfred and colleagues [23]. In our case, following the third session, sleep quality improved and fatigue gradually subsided. The patient returned to a normal sexual life after the fourth week.

To our knowledge, this is the first report to use psychotherapy only (without utilizing psychotropic drugs) in addition to conventional medications to treat OLP. A weekly consultation meeting was scheduled, during which psychoanalytic techniques were primarily used to address internal conflicts and cognitive behavior therapy was used to modify her feelings toward certain individuals and situations. Our patient was, otherwise, a young, athletic, and physically fit individual with no history of any systemic disease, familial history, or any form of drug therapy. He was a non‐smoker and non‐alcoholic. The sudden worsening of his social and economic life had caused a severe mental shock that had led to stress and ultimately depression. When the case was diagnosed, his stress and anxiety levels were significantly high as he reported an inability to sleep, exercise, go to work, or even engage in usual meaningful conversations with his spouse. In a relatively comparable case report, Song et al. reported a 32‐year‐old female OLP patient who had no apparent reaction to conventional medication. The patient's oral disease improved after switching to psychotropic medications and psychotherapy, which controlled the mood with medication and psychological counseling [24]. In a research with 62 adult OLP patients, the mean self‐perceived pain was significantly higher in the group not utilizing any stress management techniques than it was in the group doing Jacobson's Progressive Muscle Relaxation and significantly higher than the group taking the herbal sedatives [25].

## 7 Conclusion

In conclusion, this case report indicates that stress and its associated anxiety and depression have the potential to be the only possible causes for the development of erosive OLP. It was noted that the stress may have caused the disease, and the disease had worsened the stress, thus creating a closed cycle. Psychotherapy in the form of building trust and continuous communication to alleviate stress and anxiety proved to be fruitful in conjunction with the systemic and local steroids for the treatment of our patient.

## Author Contributions

**Zana Fuad Noori:** conceptualization, data curation, investigation, methodology, resources, validation, visualization, writing – original draft, writing – review and editing. **Mohammed Khalid Mahmood:** conceptualization, project administration, resources, validation, writing – original draft, writing – review and editing. **Pinar Yaseen Raof:** conceptualization, investigation, methodology, software. **Handren Ameer Kurda:** conceptualization, methodology, software, writing – original draft. **Balen Hamid Qadir:** data curation, resources, software, visualization. **Didar Anwar Abdulrahman:** conceptualization, investigation, resources, visualization. **Mohammed Aso Abdulghafor:** conceptualization, data curation, investigation, writing – original draft. **Mohammed Taib Fatih:** conceptualization, data curation, resources, software. **Basoz Khalid Hama Noori:** conceptualization, data curation, investigation, methodology.

## Consent

Written informed consent was obtained from the patient to publish this report in accordance with the journal's patient consent policy.

## Conflicts of Interest

The authors declare no conflicts of interest.

## Data Availability

The data that support the findings of this study are available on request from the corresponding author.
